# Supplementary material for: KATANIN promotes cell elongation and division to generate proper cell numbers in maize organs
Source: Nat Commun. 2026 Mar 27;17:4534. doi: 10.1038/s41467-026-71200-w (PMC13194689; doi:10.1038/s41467-026-71200-w)
Supplement: Supplementary file 1 — Supplementary Information [file 41467_2026_71200_MOESM1_ESM.pdf]

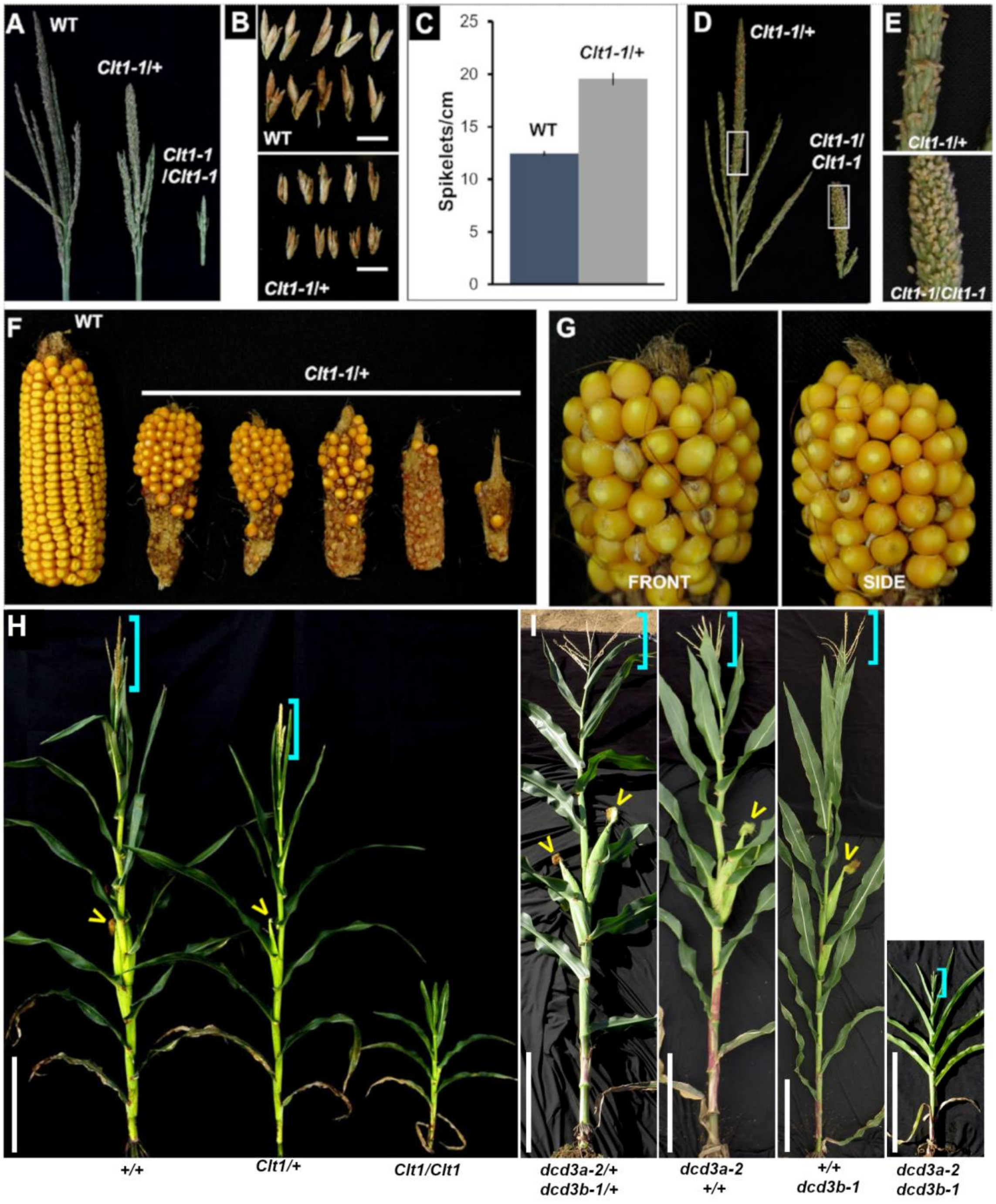

Supplementary Figure 1. Inflorescence phenotypes of *Clt1 (Clt1-1)* in the B73 background. (a-d) Comparison of tassels from the indicated genotypes. (b) Close-up of spikelets removed from the tassel. Scale bars represent 1 cm. (c) Mean spikelet density for indicated genotypes (n=20; ± standard error; Two-sided Welch's pairwise t-test, p = 3.28E-11). (d) *Clt1/*+ and *Clt1* tassels, white boxes indicate the selection for (e). (e) Spikelet phenotype from white boxed region of the tassels in panel (d). (f) Comparison of ears from the indicated genotypes. Multiple *Clt1/*+ ears shown to illustrate the range of phenotypes. (g) Front and side views of a *Clt1/*+ ear from (f), (h-i) Images of mature plants with corresponding genotypes. Yellow arrowheads indicate ears. Cyan brackets indicate visible tassels. Scale bars = 30 cm.

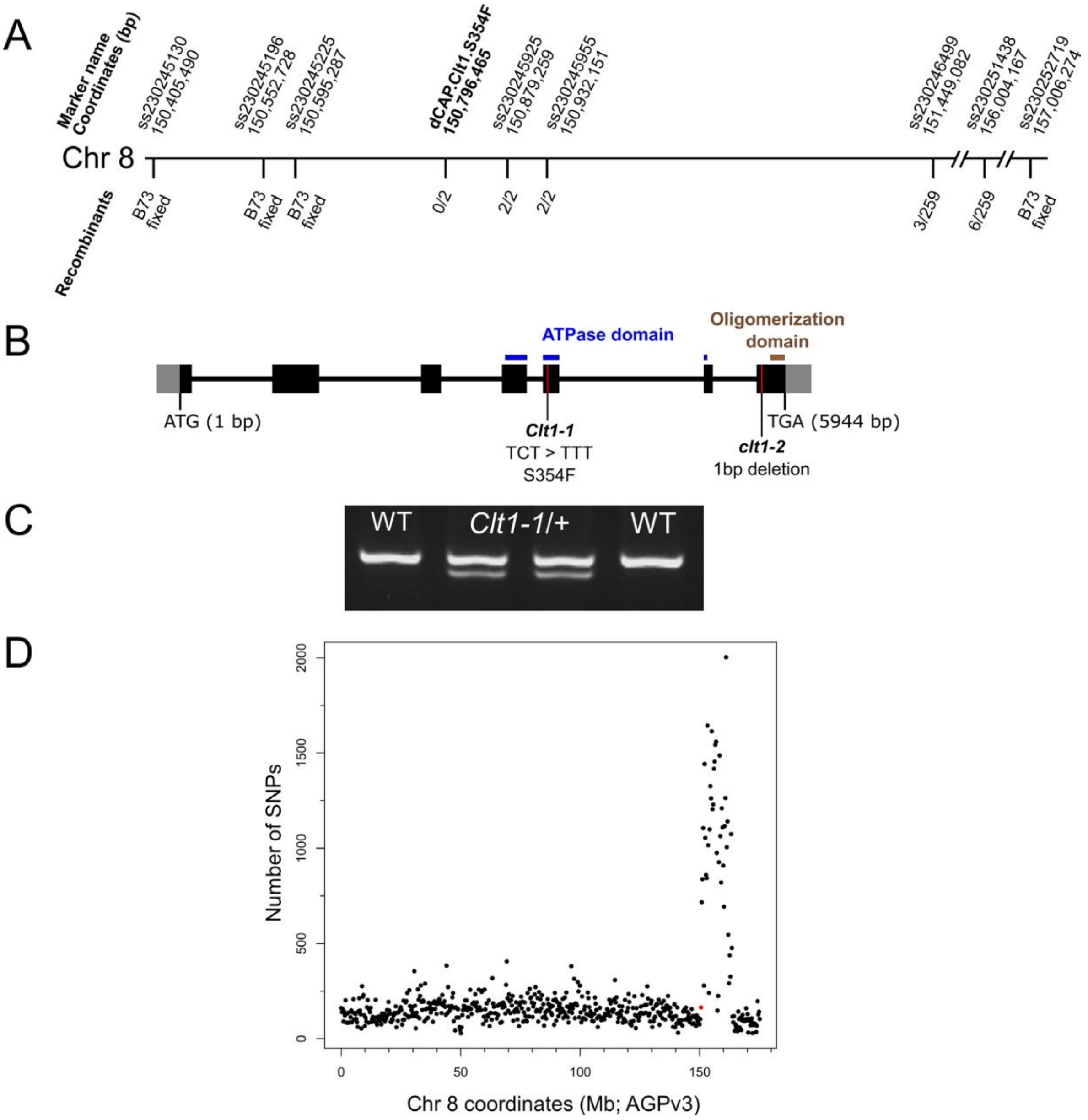

Supplementary Figure 2. Mapping *Clt1 (Clt1-1)* to the maize katanin ortholog *Dcd3b* on Chromosome 8. (a) Physical locations (B73 AGPv3) of markers used to genotype individuals from a *B73 X Clt1/+* mapping population and the number of recombinants observed at each marker. Where the number of individuals tested was only 2 instead of 259, only the plants recombinant for the outer markers were tested. (b) Gene model for *dcd3b-1* indicating locations of *Clt1-1* and *dcd3b-1*/*clt1-2*. (c) A dCAP assay directly interrogating the putative causative C to T lesion in the *dcd3b-1* locus. (d) Density of SNPs called from whole-genome sequencing of *Clt1* homozygotes, in 300 kb non-overlapping windows, with *Clt1* indicated in red.

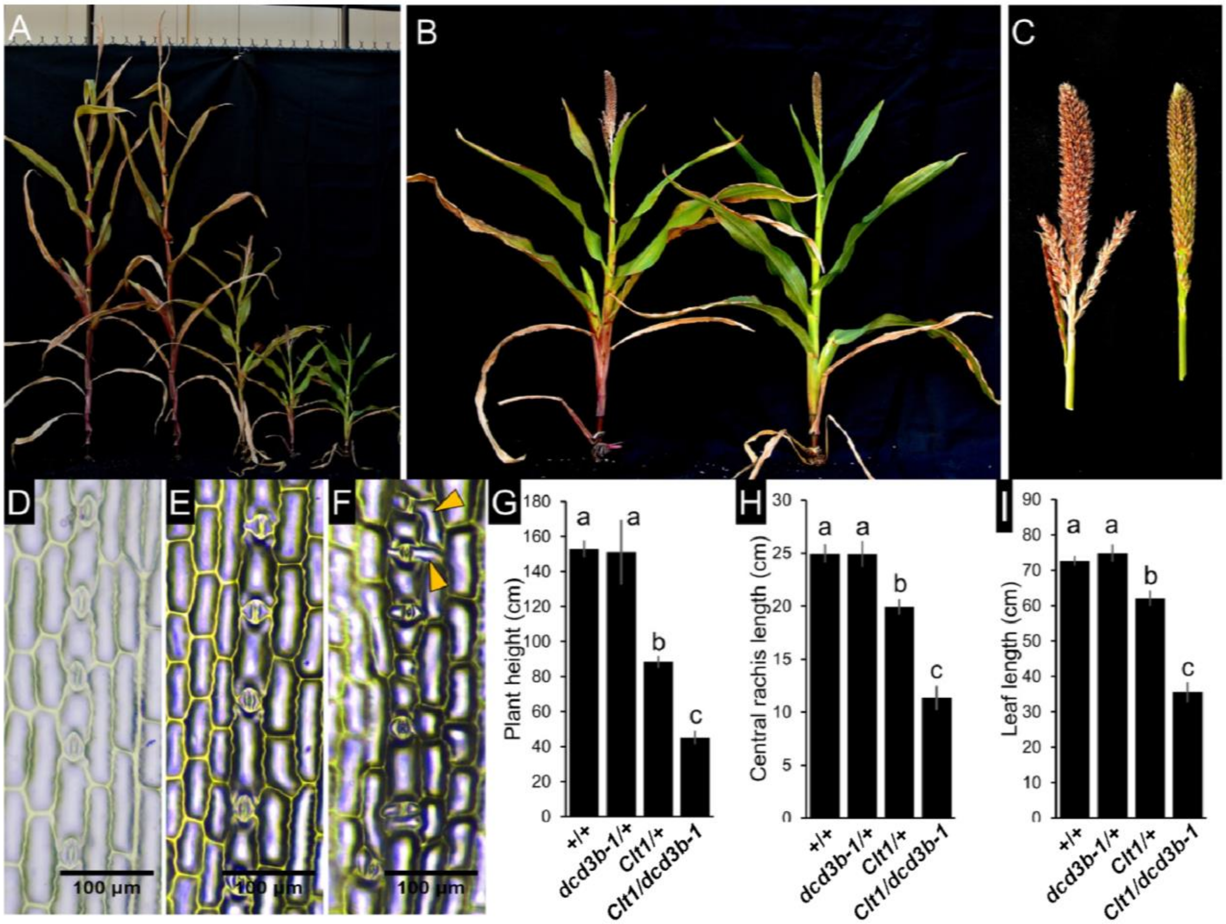

Supplementary Figure 3. *Clt1* and *dcd3b-1* interact synergistically to produce a phenotype resembling *Clt1* homozygotes. a) Mature plants +/+, *dcd3b-1/+, Clt1/+, Clt1/dcd3b-1* and *Clt1/Clt1* (left to right). (**b, c**) close-up of *Clt1/dcd3b-1* (left) and *Clt1/Clt1* (right) plants and tassels from a). (**d, e, f**) Glue impressions of epidermal cells from the third leaf of d) *dcd3b-1/*+ e) *Clt1/*+ *and* f) *Clt1/dcd3b-1* plants, yellow arrowheads indicate misoriented subsidiary cells. (**g, h, i**) Quantitative comparisons of *+/+, dcd3b-1/+, Clt1/+ and Clt1/dcd3b-1* segregating in a single family for g) plant height, h) central rachis length and i) length of the 5th leaf from the tassel. Letters represent Tukey groups calculated at the 0.05 significance level. Error bars are ± standard error. N ≥ 6 plants per genotype.

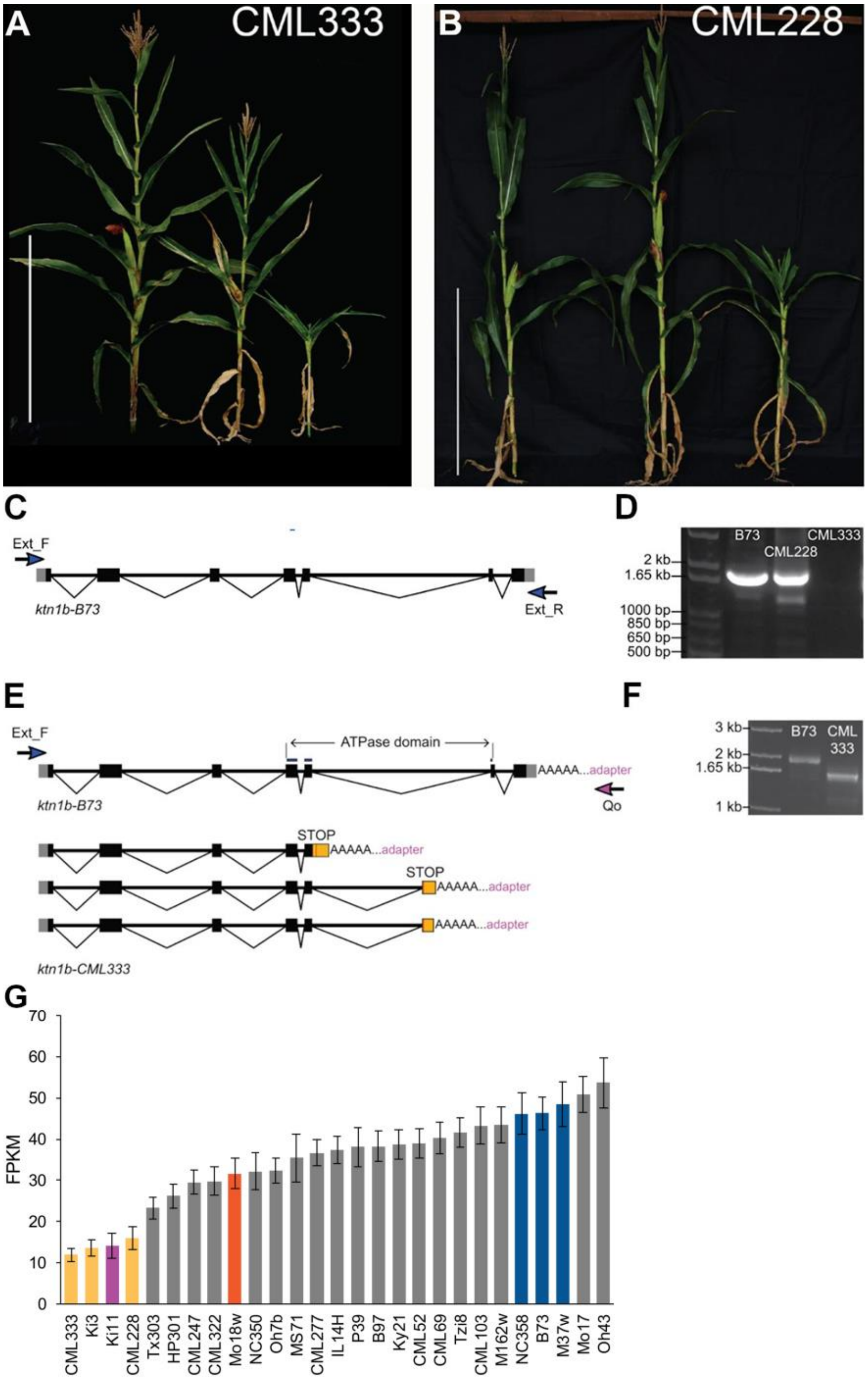

Supplementary Figure 4. Two genetic enhancers of *Clt1/+*. a) *Clt1/+ Dcd3a/Dcd3a* from B73 (left), middle is *Clt1/+ Dcd3a/dcd3a-2* (*dcd3a-2* from CML333)*, Clt1/+ dcd3a-2/dcd3a-2* (right) **b**) *Clt1/+ Dcd3a/Dcd3a* from B73 (left), middle is *Clt1/+ Dcd3a/dcd3a-1* with *dcd3a-1* from CML228, (right) *Clt1/+ dcd3a-1/dcd3a-1*. Bar in each panel is 100 cm. **c**) Gene model of *dcd3a/ktn1b* in B73. Blue arrows indicate primers binding to the 5' and 3' UTRs and used to amplify the products shown in panel (**d**). **d**) Amplification of *dcd3a (ktn1b)* cDNA from CML228 and CML333 using the primers indicated in panel (**c**). Purified gel slices were cloned and sequenced. **e**) Alternative splice forms of *dcd3a (ktn1b)* transcript

in CML333 revealed by sequencing of PCR products resulting from increasing PCR cycles from panel (**d**) and from (**f**) 3' RACE. The earliest in-frame stop codons in the retained portions of intron 5 are indicated. **f**) Amplification of CML333 *dcd3a (ktn1b)* cDNA from 3' RACE using the primers indicated in panel (**e**). **g**) Expression of *Dcd3a* at the shoot apex of 14-day old dark-grown seedlings among the NAM founder lines assessed by RNA-seq read abundance. Bar colors indicate Ki11 (magenta, *dcd3a-3*), low *Dcd3a* lines (yellow, *dcd3a-1, dcd3a-2*), Mo18w (orange), high *Dcd3a* lines (blue, including B73) and other backgrounds not examined in this work (gray). Data produced by Li et al. (2012) and analyzed by QTeller (www.qteller.com). FPKM, Fragments per kilobase of transcript per million mapped reads. Error bars indicate 95% confidence intervals.

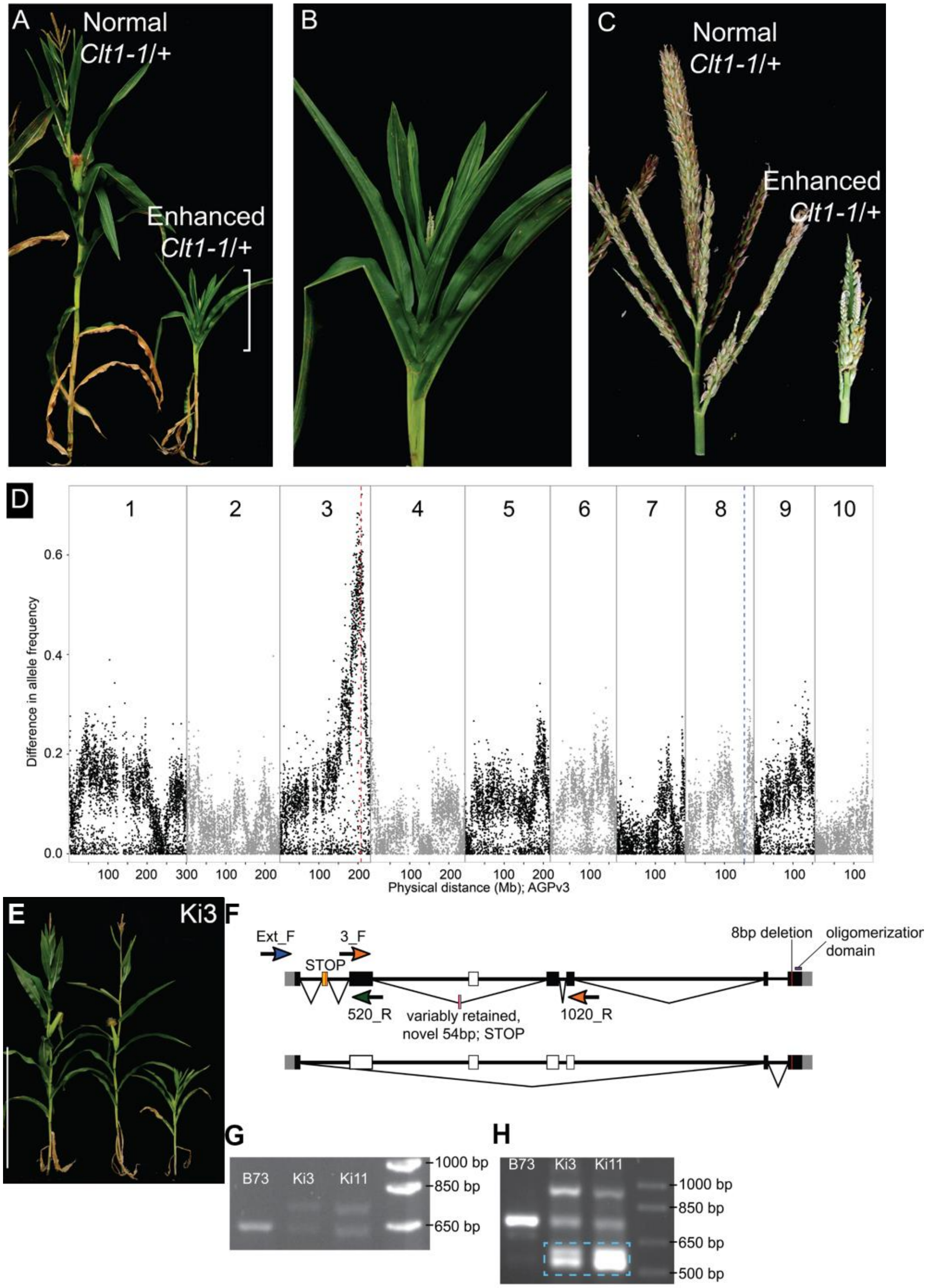

Supplementary Figure 5: Ki11 and Ki3 inbreds harbor a modifier of the mutant *Clt1* that maps to a region on Chr 3 containing *Dcd3a.* **a**) Comparison of a typical *Clt1/+ (Clt1-1/+)* heterozygote with a modified *Clt1/+* plant segregating in an F2 family derived from a cross between *Clt1/+* and Ki11. The bracket indicates the compressed upper internodes observed in the modified *Clt1/+* plants. **b**) Close-up of the compressed upper internodes in the modified plant shown in panel (**a**). **c**) Close-up of the tassels from the plants shown in panel (**a**), highlighting the reduced tassel size and poor spikelet development in the

modified *Clt1*/+ plant. **d**) BSA results calculated from the difference in allele frequencies between the normal and modified *Clt1*/+ DNA pools from 20 plants each. The dotted lines indicate the location of *Dcd3a* (red) and *Dcd3b/clt1* (blue). **e**) Left to right: *Dcd3a/Dcd3a Clt1*/+ (+/+ Clt1/+), (+/*dcd3a-3* Clt1/+) and (*dcd3a-3 Clt1*/+). **f**) Alternative spliceforms of *dcd3a-3* transcript from Ki3 and Ki11 identified by sequencing. Note that the 54 bp (pink) was identified from the Ki3 sequence while the spliceform missing exons 2-5 (bottom) was identified by sequencing in Ki11 but PCR suggests that the features are present in both Ki3 and Ki11. In-frame stop codons caused by the partial retention of intron 1 and the 54 bp sequence are indicated. An 8 bp deletion occurs in the final exon in both lines causing a frameshifted sequence in the oligomerization domain. (**g, h**) Amplification of *dcd3a-3* cDNA using primers indicated in panel (**f**) corroborate aberrant spliceforms shown in panel (**g**). **h**) The blue dashed box indicates products corresponding to excision of exon 3 and variable retention of the 54 bp novel sequence.

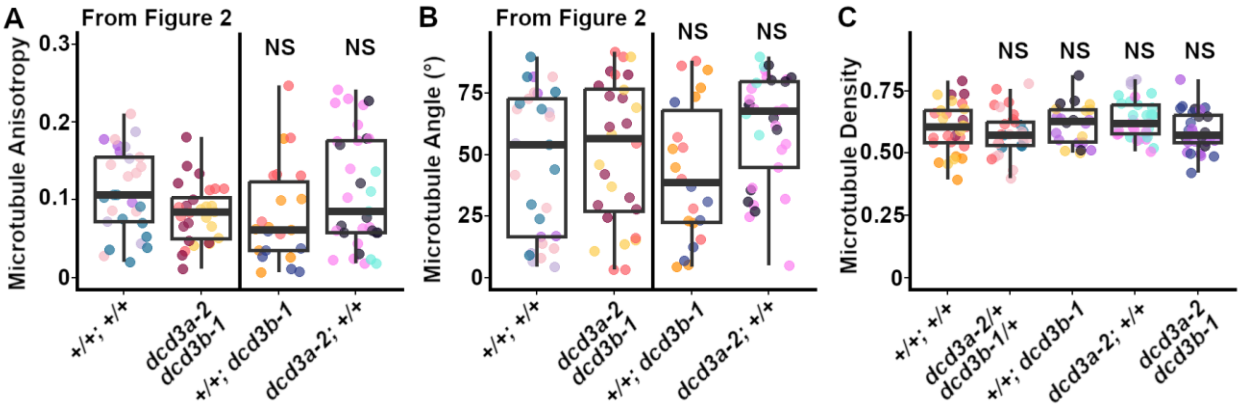

Supplementary Figure 6. Microtubule anisotropy, density, and average angle against the growth axis is not significantly different in *katanin* mutant combinations. a) Microtubule anisotropy, **b)** angle against the growth axis of the leaf, and **c)** density was measured from at least 22 cells from at least 3 plants per genotype. Each color = an individual plant, each dot = a cell. For **(a)** and **(b)**, microtubule anisotropy and angle for +/+*;* +/+ and *dcd3a-2 dcd3b-1* was taken from Figure 2. A pairwise Wilcoxon comparison (with Bonferroni correction 0.05/5=0.01) was done between +/+*;* +/+ and other alleles shown in a-c. Boxplots show median and quartiles with whiskers ≤1.5X the interquartile range. The exact plant number, cell number and p-value is in Source Data.

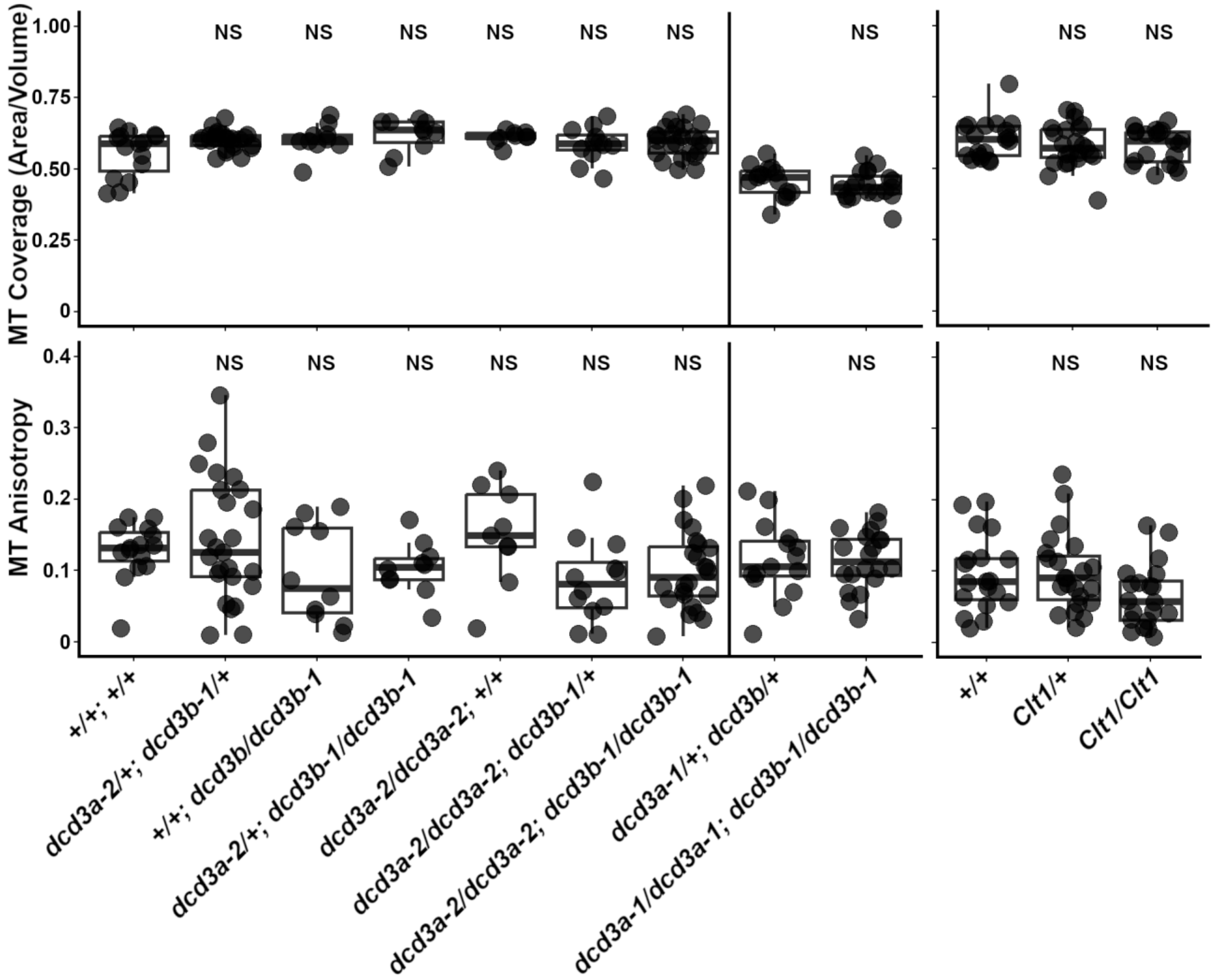

Supplementary Figure 7. Microtubule coverage and anisotropy of cells selected for measuring severing in the elongation zone of the emerging leaf are not significantly different. Microtubule coverage was measured using the BoneJ plugin (see Materials and Methods). The average microtubule coverage for wild type (+/+ +/+) (0.554 ± 0.021 SE), *dcd3a-2/+ dcd3b-1/+* (0.601 ± 0.006 SE; p-value = 0.008), and *dcd3a-2 dcd3b-1* (0.593 ± 0.011 SE; p-value = 0.02). The average microtubule coverage for *dcd3a-1/+ dcd3b-1/+* and *dcd3a-1 dcd3b-1* was 0.460 ± 0.014 SE and 0.444 ± 0.011 SE, respectively (p-value = 0.374). The average microtubule coverage for wild-type (+/+) *clt1/clt1* (0.601 ± 0.016 SE), *Clt1/+* (0.579 ± 0.016 SE; p-value = 0.29), and *Clt1/Clt1* (0.580 ± 0.014 SE; p-value = 0.34). Microtubule anisotropy was obtained using FibrilTool (see Methods). The average microtubule anisotropy for *+/+; +/+* (0.128 ± 0.010 SE), *dcd3a-2/+; dcd3b-1/+* (0.142 ± 0.017 SE; p-value = 0.45), and *dcd3a-2 dcd3b-1* (0.099 ± 0.011 SE; p-value = 0.16). The average microtubule anisotropy for *dcd3a-1/+ dcd3b-1/+* and *dcd3a-1 dcd3b-1* was 0.115 ± 0.014 SE and 0.142 ± 0.009 SE, respectively (p-value = 1). The average microtubule anisotropy for +/+ (*clt1/clt1*) (0.097 ± 0.012 SE), *Clt1/+* (0.098 ± 0.011 SE; p-value = 1), and *Clt1/Clt1* (0.66 ± 0.010 SE; p-value = 0.06). P-values were obtained from Two-sided pairwise t-tests (with Bonferroni correction .05/6=0.008 or .05/2=0.025). Boxplots show median and quartiles with whiskers ≤1.5X the interquartile range. Exact cell numbers, plant numbers and p-values are in Source Data.

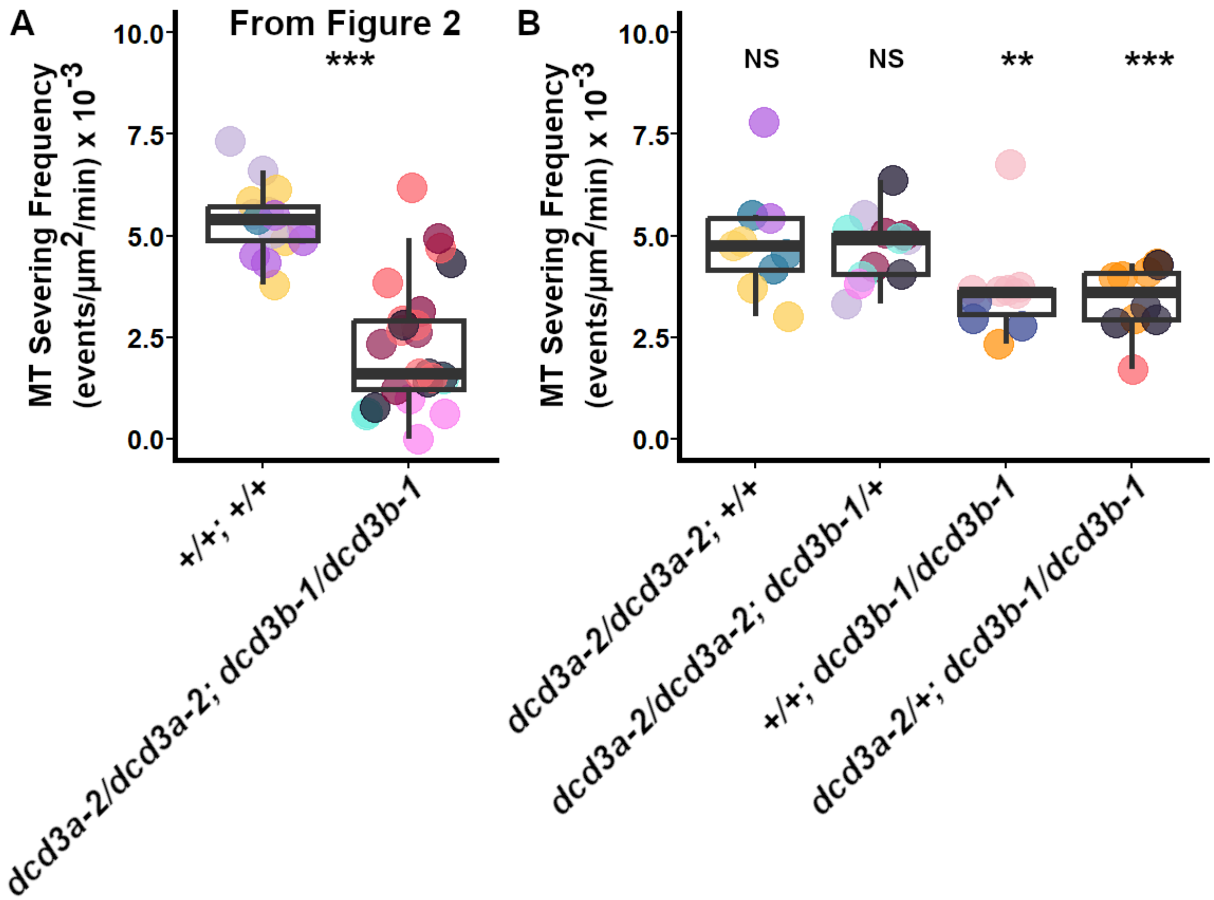

Supplementary Figure 8. Microtubule severing frequency (events/µ^2/min) X 10^{-3} in additional *katanin* mutant allele combinations. a) Microtubule severing frequency for +/+; +/+ and *dcd3a-2/dcd3a-2 dcd3b-1/dcd3b-1* taken from Figure 2. Pairwise comparison (with Bonferroni correction .05/4=0.0125) was done between +/+*;* +/+ and other alleles shown in (**b**). **b)** Microtubule severing frequency in *dcd3a-2/dcd3a-2;* +/+ averaged 4.9E-03 events/µm^2/min ± 0.04E-03 from n = 9 cells from 3 plants, p = 0.25; *dcd3a-2/dcd3a-2; +/dcd3b-1* averaged 4.7E-03 events/µm^2/min ± 0.02E-03 SE from n = 12 cells from 6 plants, p = 0.09; +/+*; dcd3b-1/dcd3b-1* averaged 3.7E-03 events/µm^2/min ± 0.04E-03 SE from n = 10 cells from 4 plants, p < 0.0001; *+/dcd3a-2, dcd3b-1/dcd3b-1* average 3.5E-03 events/µm^2/min ± 0.02E-03 SE from n = 10 cells from 3 plants, p = 0.00002. Each color = an individual plant. Boxplots show median and quartiles with whiskers ≤1.5X the interquartile range. Exact cell numbers, plant numbers and p-values are in the Source Data.

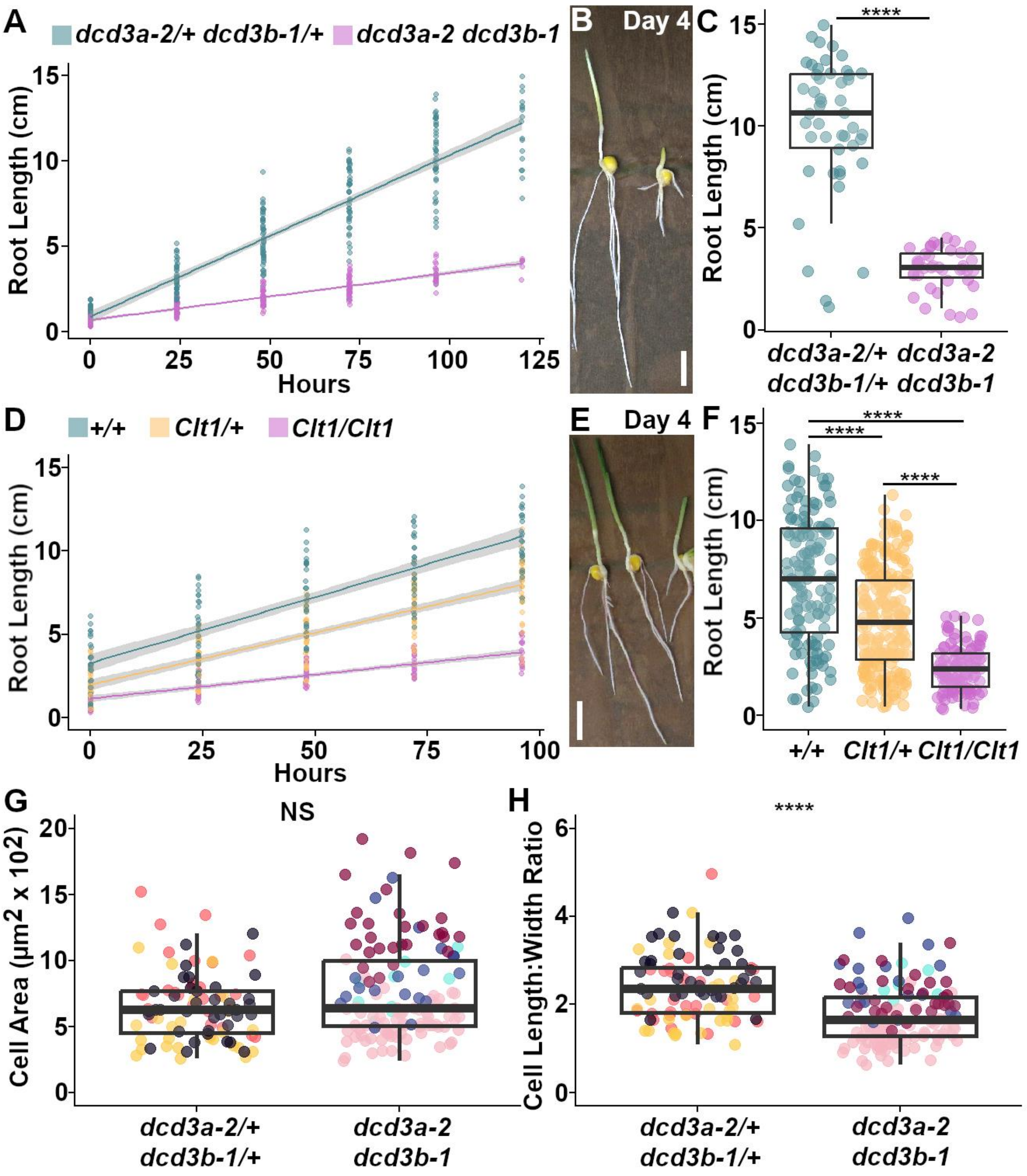

Supplementary Figure 9. The *katanin* mutants have decreased root growth over time. a) For the first five days after germination, *katanin* mutants had decreased linear root growth rate when compared to wild-type siblings (*dcd3a/+ dcd3b-1/+*: 2.4 cm/day ± 0.07 cm/day; *dcd3a-2 dcd3b-1*: 0.48 cm/day ± 0.02 cm/day). **b)** Representative *dcd3a-2/+ dcd3b-1/+* (left) and *dcd3a-2 dcd3b-1* (right) seedlings 4 days after germination. Scale bar = 2 cm. **c)** Root length of *dcd3a-2/+ dcd3b-1/+* (10.3 cm ± 3.1 cm; n = 46 seedlings) and *dcd3a-2 dcd3b-1* (3.0 cm ± 1.0 cm; n = 39 seedlings) at 4 days post germination. Two-sided Welch's pairwise t-test p-value = 3.7e-20 **d)** Linear root growth rate for wild type (1.8 cm/day ± 0.2

cm), *Clt1*/+ (1.5 cm/day ± 0.1 cm), and *Clt1/Clt1* (0.7 cm/day ± 0.1 cm). **e)** Representative wild-type (left), *Clt1*/+ (middle), and *Clt1/Clt1* (right) seedlings at 4 days post germination. Scale bar = 2 cm. **f**) Root length of wild type (10.3 cm ± 2.3 cm; n = 27 plants), *Clt1*/+ (7.6 cm ± 2.2 cm; n = 44 plants), and *Clt1/Clt1* (3.6cm ± 1.1 cm; n = 22 plants) at 4 days after germination. **** asterisk indicates p-value ≤ 4e-12 from a two-sided pairwise t-test with Bonferroni correction. **g**) Cell area (μm^2) of root epidermal cells in the elongation zone just below root hairs in *dcd3a-2/+ dcd3b-1/+* (average 652 ± 27 μm^2 at 0.5 cm from root tip, n = 91 cells) and *dcd3a-2 dcd3b-1* (average 781 ± 36 μm^2 from ~0.2 cm from root tip, n = 129 cells). Wilcoxon rank sum test p-value = 0.05. **h**) Cell length:width ratio of root epidermal cells in *dcd3a-2/+ dcd3b-1/+* (2.41 ± 0.08) and *dcd3a-2 dcd3b-1* (1.77 ± 0.06). Wilcoxon rank sum test p-value = 3.8e-10. Boxplots show median and quartiles with whiskers ≤1.5X the interquartile range. Exact cell numbers, plant numbers and p-values are in Source Data.

	Leaf Number	Wild type (*dcd3a-2/+ dcd3b-1/+*)	Mutant (*dcd3a-2 dcd3b-1*)	Exact p-value
**Leaf Area (cm^2) ± SE**	1	5.7 ± 0.3	1.6 ± 0.2	0.0002***
	5	145 ± 19	51 ± 8	0.006*
	8	473 ± 44	148 ± 9	0.0147*
**Total Approximate Cells ± SE**	1	4.2E+05 ± 3.4E+04	1.3E+05 ± 1.1E+04	0.002**
	5	8.6E+06 ± 1.2E+06	4.2E+06 ± 7.7E+05	0.034*
	8	3.9E+07 ± 6.0E+06	1.4E+07 ± 7.3E+05	0.018**

Supplementary Table 1. The *dcd3a-2 dcd3b-1* double mutants have smaller leaves with fewer cells than wild-type siblings. Leaf Area (cm^2) ± standard error is shown. Approximate total cell count ± standard error is shown. N ≥ 3 plants per genotype per leaf. P-values from a two-tailed Student's t-test. Exact cell numbers, plant numbers and p-values are in the Source Data.

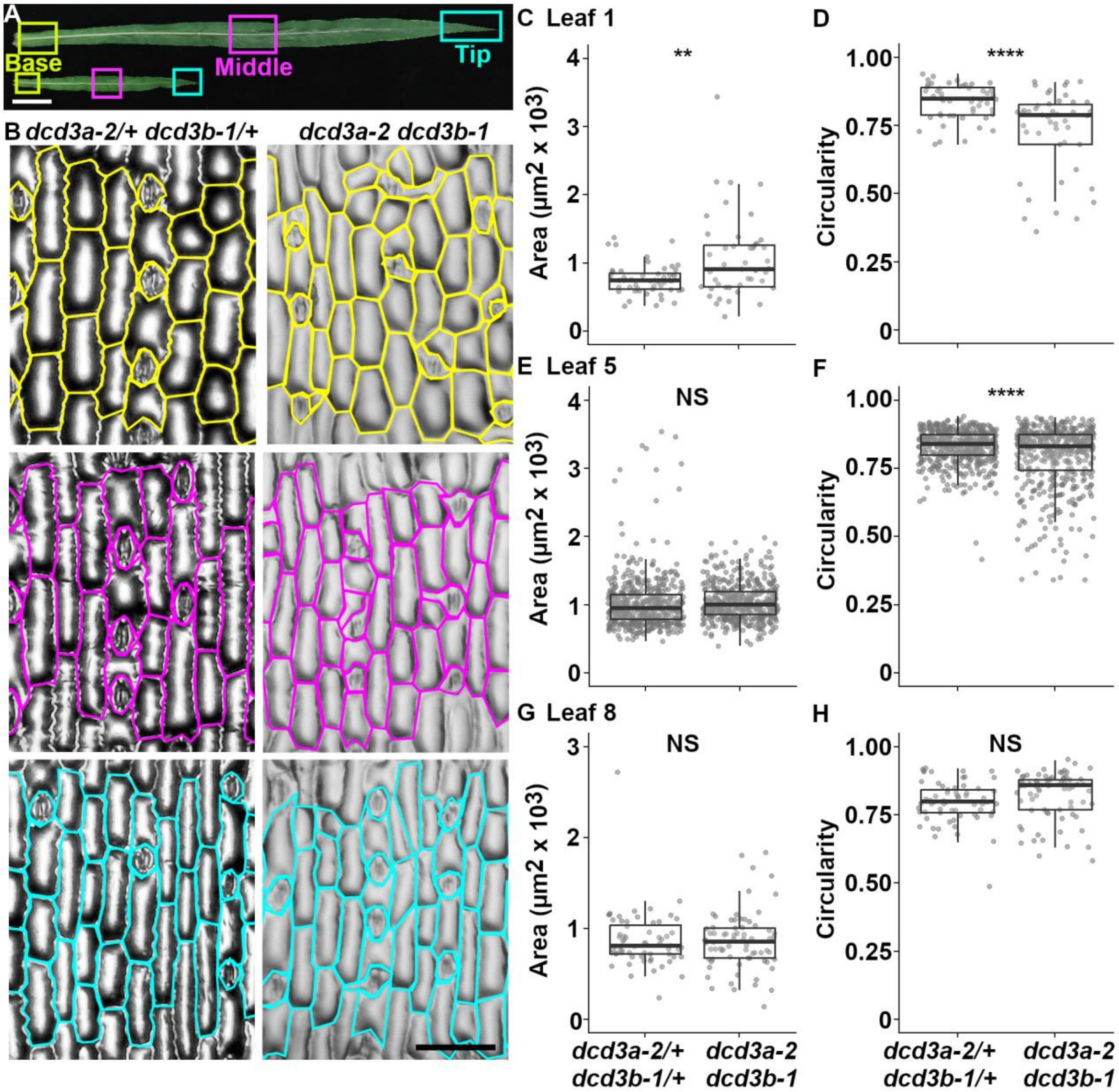

Supplementary Figure 10. Area and circularity of stomatal complexes in *dcd3a-2 dcd3b-1* double mutants and wild-type siblings. (a) Base (yellow), middle (magenta), and tip (cyan) regions of leaf 1, 5 (shown here), and 8 were taken for glue impressions. Scale bar = 5 cm. (b) Representative examples of pavement cell and stomatal complex outlines from *dcd3a-2/+ dcd3b-1/+* and *dcd3a-2 dcd3b-1* glue impressions from the base (yellow outlined), middle (magenta outlines), and tip (cyan outlines) regions of the leaf. All images were taken at the same magnification. Scale bar = 100 µm. (c) Stomatal complex areas from leaf 1. (d) Circularity of stomatal complexes from leaf 1. (d) Stomatal complex areas from leaf 5 (f) Circularity of stomatal complexes from leaf 5. (g) Stomatal complex areas from leaf 8 (h). Circularity of stomatal complexes from leaf 8. Supporting data in Supplementary Table 2 and Source Data. Two-sided Welch's pairwise t-test p-value < .01=**; < .00005= ****. Boxplots show median and quartiles with whiskers ≤1.5X the interquartile range.

**Measured Feature**	**Leaf #**	**Wild type (*dcd3a-2/+ dcd3b-1/+*)**	**Mutant (*dcd3a-2 dcd3b-1*)**
Total Pavement Cells Measured	1	n = 153 from 4 plants	n = 404 from 3 plants
	5	n = 1736 from 4 plants	n =2117 from 3 plants
	8	n = 204 from 3 plants	n = 336 from 3 plants
Median Pavement Cell Area (μm^2) ± SE	1	2781 ± 112 μm^2	1557 ± 46 μm^2 *** p-value 5.0e-24
	5	2957 ± 31 μm^2	2099 ± 17 μm^2 **** p-value 1.7e-155
	8	2554 ± 61 μm^2	1719 ± 40 μm^2 **** p-value 1.3e-24
Median Pavement Cell Height (μm) ± SE	1	125 ± 3.6 μm	64 ± 1.5 μm **** p-value 2e-37
	5	113 ± 0.9 μm	80 ± 0.6 μm ****p-value 2.8e-182
	8	94 ± 2.3 μm	68 ± 1.3 μm **** p-value 1.9e-129
Median Pavement Cell Width (μm) ± SE	1	23.7± 0.5 μm	24.7 ± 0.4 μm NS p-value 0.18
	5	26.6 ± 0.2 μm	25.9 ± 0.1 μm **** p-value 3e-16
	8	26 ± 0.5 μm	26 ± 0.4 μm NS p-value 0.5
Total Stomatal Complexes Measured	1	51 from 4 plants	46 from 3 plants
	5	496 from 4 plants	465 from 3 plants
	8	64 from 3 plants	72 from 3 plants
Median Stomatal Complex Area (μm^2) ± SE	1	745 ± 31.7 μm^2	915 ± 88.3 μm^2 ** p-value 1.6e-3
	5	953 ± 18 μm^2	1,005 ± 13 μm^2 NS p-value 0.44
	8	811± 39 μm^2	858± 38 μm^2 NS p-value 0.85
Median Stomatal Complex Circularity± SE	1	0.85 ± 0.01	0.79 ± 0.02 **** p-value 4.8e-5
	5	0.84 ± 0.003	0.83 ± 0.005 **** p-value 3.7e-8
	8	0.80 ±0.01	0.86 ± 0.01 NS p-value 0.1

Supplementary Table 2. The *dcd3a-2 dcd3b-1* double mutants have smaller cells. Pavement cell and stomatal complex measurements for *dcd3a-2/+ dcd3b-1/+* and *dcd3a-2 dcd3b-1* leaves 1, 5, and 8. Two-sided Welch's pairwise t-test p-value as indicated, NS = not significant. These data are used in Figure 3 and Supplementary Figure 10.

**Measured time**	**Wild type** (*dcd3a-2/+ dcd3b-1/+*)	**Mutant** (*dcd3a-2 dcd3b-1*)
Median Metaphase Time (mins ± SE)	30 ± 7	30 ± 5 p-value = 0.065
Median Telophase Time (mins ± SE)	50 ± 3	60 ± 4 p-value 0.06
Mean Phragmoplast Expansion Rate (µm/min ± SE)	0.31 ± 0.02	0.30 ± 0.02 p-value 0.27
Median Cell Division Time (mins ± SE)	90 ± 9	110 ± 7 p-value 0.87

Supplementary Table 3. The *dcd3a-2 dcd3b-1* double mutants have similar cell division times as wild-type siblings. Data was obtained from n = 69 cells from 6 *dcd3a-2/+ dcd3b-1/+* plants and n = 48 cells from 8 *dcd3a-2 dcd3b-1* plants. Two-sided Wilcoxon rank sum test p-values were > 0.05 for all measurements.

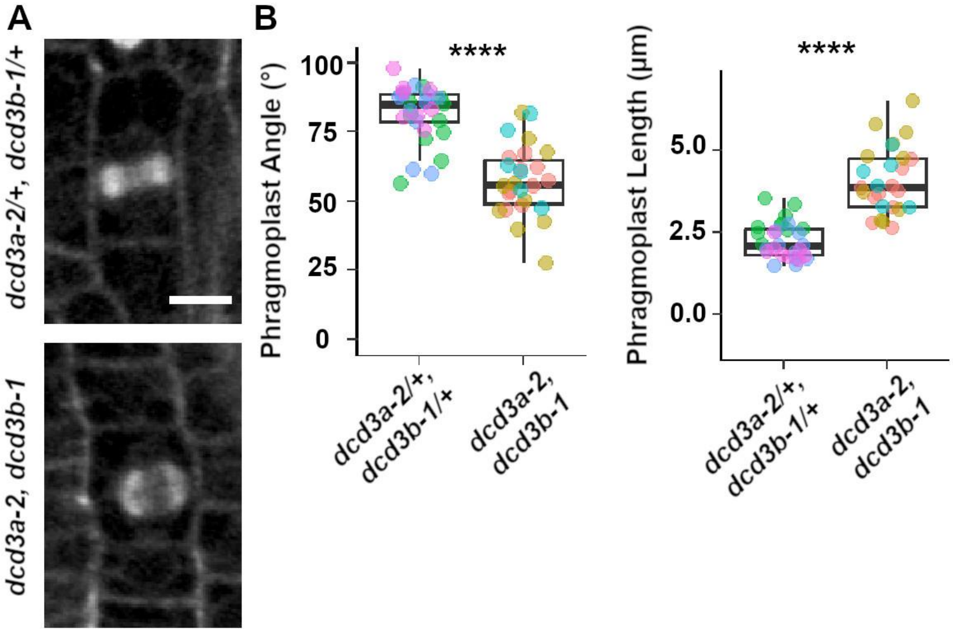

Supplementary Figure 11. *dcd3a-2 dcd3b-1* mutants have decreased phragmoplast angles and increased lengths when compared to wild-type siblings. **a**) Representative micrographs comparing phragmoplasts in wild-type and mutant cells. 3 plants per genotype were analyzed. **b**). Phragmoplast angle (°) and Phragmoplast length (μm). Each dot = one cell. Each color = a plant. n= 29 for *dcd3a-2/+ dcd3b-1/+* and n = 26 cells for *dcd3a-2 dcd3b-1*; Wilcoxon rank sum test p-value = 1.139E-10 = ****, Wilcoxon test p-value = 1.138E-10 = ****. Scale bar = 10 μm. Boxplots show median and quartiles with whiskers ≤1.5X the interquartile range.

**Mitotic Stage**	**Wild-type**<br>(*dcd3a-2/+ dcd3b-1/+)*	**Mutant**<br>(*dcd3a-2 dcd3b-1*)
Preprophase/prophase	54% (101)	59% (114) NS p-value 0.35
Metaphase/anaphase	12.8% (24)	9.8% (19) NS p-value 0.42
Telophase/cytokinesis	33.2% (62)	31.1% (60) NS p-value 0.74
Total mitotic cells	187	193

Supplementary Table 4. The *dcd3a-2 dcd3b-1* double mutants have similar proportions of late G2 and mitotic cells compared to wild-type siblings. Data was obtained from three plants of each genotype for wild type plants (*dcd3a-2/+ dcd3b-1/+*) and mutant plants (*dcd3a-2 dcd3b-1*). Two-sided Fisher's exact test used for p-values. Data for Fig. 4b. This data is a subset of the data from Supplementary Table 5.

**Stage**	**Wild type** (*dcd3a-2/+ dcd3b-1/+*)	**Mutant** (*dcd3a-2 dcd3b-1)*	**Exact p-value**
Interphase	86% (1179)	91% (2039)	2.9e-10***
Preprophase/prophase	7.4 % (101)	5.1% (114)	0.02*
Metaphase/anaphase	1.8% (24)	0.9% (19)	0.03*
Telophase/cytokinesis	4.5% (62)	2.7% (60)	0.008**
Total dividing cells	13.7% (187)	8.6% (193)	2.5e-5***
Total cells	1366	2232	

Supplementary Table 5: The *dcd3a-2 dcd3b-1* double mutants have less dividing cells compared to wild-type siblings. Data was obtained from three plants of each genotype for wild type plants (*dcd3a-2/+ dcd3b-1/+*) and mutant plants (*dcd3a-2 dcd3b-1*). Two-sided Fisher's exact test p-values are shown. Data for Figure 4B and Supplementary Table 4.

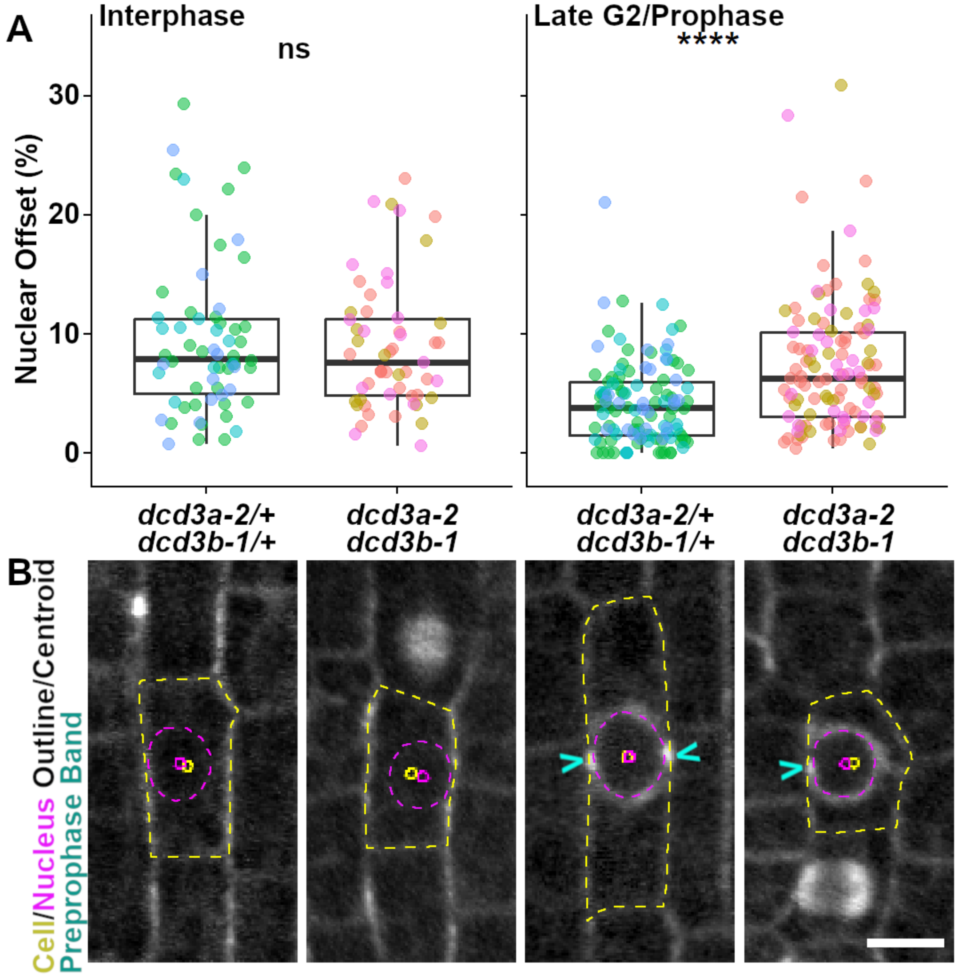

Supplementary Figure 12. *dcd3a-2 dcd3b-1* mutants have more offset nuclei during prophase when compared to wild-type siblings. (**a**). Boxplots showing normalized nuclear offset (%) from the cell center for cells in interphase and G2/prophase. For interphase: *dcd3a-2/+ dcd3b-1/+*: 9.5 ± 0.8, n = 62 cells from 3 plants; *dcd3a-2 dcd3b-1*: 8.9 ± 0.7, n = 55 cells from 3 plants. For G2/prophase: *dcd3a-2/+ dcd3b-1/+*: 4.2 ± 0.3, n = 117 cells from 3 plants; *dcd3a-2 dcd3b-1*: 7.4 ± 0.5, n = 113 cells from 3 plants. Different colored dots = different plants. Two-sided Wilcoxon test p-value = 4.2E-07 = ****, Two-sided Wilcoxon test p-value = 0.7 = NS. Boxplots show median and quartiles with whiskers ≤1.5X the interquartile range. (**b**). Representative micrographs of *dcd3a-2/+ dcd3b-1/+* and *dcd3a-2 dcd3b-1* during interphase and G2/prophase. The cell (yellow) and nucleus (magenta) outline with their respective centroids are shown. The preprophase band is marked by the cyan arrows. Scale bar = 10 µm.

	**Normal (n=17 cells)**	**Uneven (n=23 cells)**	**One-Sided (n=7 cells)**
**Median Metaphase Time (mins ± SE)**	30 ± 8	30 ± 6<br>NS p-value 0.26	70 ± 20<br>NS p-value 0.6
**Median Telophase Time (mins ± SE)**	40 ± 4	60 ± 7<br>* p-value 0.02	70 ± 9<br>* p-value 0.02
**Median Total Time (mins ± SE)**	90 ± 9	110 ± 9<br>NS p-value .08	160 ± 20<br>* p-value 0.02

Supplementary Table 6. Division times are longer in cells with aberrant PPBs in the *dcd3a-2 dcd3b-1* double mutant. Data was obtained from 8 *dcd3a-2 dcd3b-1* plants. Wilcoxon rank sum test with Bonferroni correction 0.05/2 = 0.025 was performed to compare uneven and one-sided PPBs to cells with normal PPBs. *<0.025, NS >0.025.

Primer Name	Primer Sequence	Use
3kat_177F	CGAAACCAAACCAAACCACTCTCC	For *dcd3a-1* PCR amplification, followed by SspI digestion
3kat_565R	GGATAGACCCAACAACATAGCCAAC	For *dcd3a-1* PCR amplification, followed by SspI digestion
3kat_1CFOR	CTTGCTCAAGAGCCCAGC	For *dcd3a-2* genotyping
3kat_rev1a	CGAGACGCGACACTCGC	For *dcd3a-2* genotyping
3kat_13671rev	AGGAGGTGGTGCATAGCC	For *dcd3a-2* genotyping
Clt1-1F	TAATTTTCCTAGATGTTGCTCGTGT	For *Clt1* PCR amplification, followed by DraI digestion
Clt1-1R	ACCATCAATTTGCACTAGAAGTTTA	For *Clt1* PCR amplification, followed by DraI digestion
8kat_3655F	GATGTTGCTCGTGTCTACAAGTC	For *dcd3b-1* PCR amplification, followed by MspI digestion
8kat_in5aR	TGAACCTATGCATATTCCAGG	For *dcd3b-1* PCR amplification, followed by MspI digestion
ZmTUB-alpha_FP1	GCAAGGTTTCGATTTCCGTA	For YFP-TUBULIN genotyping
ZmTUB-alpha_RP1	GGTTTCGGGTGATCCCTATT	For YFP-TUBULIN genotyping
GFP5FOR	GCGACGTAAACGGCCACAAGTTCAG	For CFP-TUBULIN genotyping
TubB3433R	CGGAAGCAGATGTCGTAGAGC	For CFP-TUBULIN genotyping
TAN LSP1	ACGACCGTTAGCACAGAACC	For TAN1-YFP genotyping
GFP5 Rev	CTGAACTTGTGGCCGTTTACGTCGC	For TAN1-YFP genotyping
Ext_F	CGTTCTCTCCATCGAACTGGC	For DCD3A amplification
Ext_R	CAGCGGTCAGCGGATATCAA	For DCD3A amplification
520_R	GTTAGAGGCAGTTGATGATTTAACC	For *dcd3a-3* transcript amplification
3_F	AACCGTTGGACGAGTATCCA	For *dcd3a-3* transcript amplification
1020_R	GACTCATGTTCACCAGATGCT	For *dcd3a-3* transcript amplification
Q_o	CCAGTGAGCAGAGTGACG	For *dcd3a-2* transcript amplification

Supplementary Table 7. Primers used in this study.
