# Supplementary material for: KATANIN promotes cell elongation and division to generate proper cell numbers in maize organs
Source: Nat Commun. 2026 Mar 27;17:4534. doi: 10.1038/s41467-026-71200-w (PMC13194689; doi:10.1038/s41467-026-71200-w)
Supplement: Supplementary file 2 — Description of Additional Supplementary Information [file 41467_2026_71200_MOESM2_ESM.pdf]

Description of Additional Supplementary Files

File Name: Supplementary Movie 1
Description: Time-lapse of a cell with a normal PPB undergoing symmetric cell division. Microtubules = green, TAN1-YFP = magenta, Scale bar = 5 µm. Time stamp format is hours:minutes:seconds.

File Name: Supplementary Movie 2
Description: Time-lapse of a cell with an uneven PPB undergoing symmetric cell division. Microtubules = green, TAN1-YFP = magenta, Scale bar = 5 µm. Time stamp format is hours:minutes:seconds.

File Name: Supplementary Movie 3
Description: Time-lapse of a dcd3a-2 dcd3b-1 cell with an one-sided PPB undergoing symmetric cell division. Microtubules = green, TAN1-YFP = magenta, Scale bar = 10 µm. Time stamp format is hours:minutes:seconds.

File Name: Supplementary Movie 4
Description: Time-lapse of a dcd3a-2 dcd3b-1 cell with a misoriented PPB undergoing symmetric cell division. Microtubules = green, TAN1-YFP = magenta, Scale bar = 5 µm. Time stamp format is hours:minutes:seconds.
